# Electrospun Poly(L-lactide)/Poly(*ε*-caprolactone) Blend Nanofibrous Scaffold: Characterization and Biocompatibility with Human Adipose-Derived Stem Cells

**DOI:** 10.1371/journal.pone.0071265

**Published:** 2013-08-26

**Authors:** Liang Chen, Yi Bai, Guiying Liao, Ejun Peng, Bolin Wu, Yuxi Wang, Xiaoyong Zeng, Xiaolin Xie

**Affiliations:** 1 Department of Urology, Tongji Hospital, Tongji Medical College, Huazhong University of Science and Technology, Wuhan, Hubei, China; 2 Department of Oral Implantology, School and Hospital of Stomatology, Wuhan University, Wuhan, Hubei, China; 3 School of Material Science and Chemistry Engineering, China University of Geosciences (Wuhan), Wuhan, Hubei, China; 4 Hubei Key Laboratory of Materials Chemistry and Service Failure, School of Chemistry and Chemical Engineering, Huazhong University of Science and Technology, Wuhan, Hubei, China; Instituto Butantan, Brazil

## Abstract

The essence of tissue engineering is the fabrication of autologous cells or induced stem cells in naturally derived or synthetic scaffolds to form specific tissues. Polymer is thought as an appealing source of cell-seeded scaffold owing to the diversity of its physicochemical property and can be electrospun into nano-size to mimic natural structure. Poly (L-lactic acid) (PLLA) and poly (ε-caprolactone) (PCL) are both excellent aliphatic polyester with almost “opposite” characteristics. The controlling combination of PLLA and PCL provides varying properties and makes diverse applications. Compared with the copolymers of the same components, PLLA/PCL blend demonstrates its potential in regenerative medicine as a simple, efficient and scalable alternative. In this study, we electrospun PLLA/PCL blends of different weight ratios into nanofibrous scaffolds (NFS) and their properties were detected including morphology, porosity, degradation, ATR-FTIR analysis, stress-stain assay, and inflammatory reaction. To explore the biocompatibility of the NFS we synthesized, human adipose-derived stem cells (*h*ASCs) were used to evaluate proliferation, attachment, viability and multi-lineage differentiation. In conclusion, the electrospun PLLA/PCL blend nanofibrous scaffold with the indicated weight ratios all supported *h*ASCs well. However, the NFS of 1/1 weight ratio showed better properties and cellular responses in all assessments, implying it a biocompatible scaffold for tissue engineering.

## Introduction

Tissue engineering is a thriving cross-disciplinary field which is widely recognized as the most promising access for diseased or damaged tissues [1], [2]. The essential strategy of tissue engineering is the application of implanted autologous cells or induced stem cells to form specific tissues in naturally derived or synthetic scaffold which offers a temporary shelter. The successful scaffold depends on taking the place of natural extracellular matrix (ECM) to guide cellular responses including proliferation, adhesion, migration, and differentiation [3], [4].

With the urge of environmental concern, synthetic polymers have attracted more attention than natural materials [5]. The polymeric scaffolds are versatile in manipulating physicochemical properties such as degradation, surface charge and mechanical strength [6]. They are usually made into nano-sized (tens to hundreds of nanometers) and three-dimensional structure to simulate natural ECM [7]. To achieve this goal, electrospinning is a convenient and inexpensive way. Up to date, the various nanofibrous scaffolds (NFS) synthesized by electrospinning are regarded as candidate scaffolds for ECM replacement in regenerative medicine [8], [9].

As the representative of polymers, aliphatic polyesters, ranging from poly (L-lactic acid) (PLLA), poly (ε-caprolactone) (PCL), poly (glycolic acid) (PGA) and their composites, have been developed to replace ECM due to their excellent biocompatibility [3], [5], [6], [10]–[13]. Among these aliphatic polyester members, PLLA and PCL, which are both FDA-approved, interestingly show the almost “opposite” properties. PLLA is brittle with high degradation and better tensile strength, while PCL is flexile with low degradation and better toughness [5]. The varying combination of glassy PLLA and rubbery PCL makes their use application-dependent.

Till now, the electrospun copolymers of PLLA and PCL have been extensively investigated [3], [14]–[16]. Compared with relatively intricate copolymerization method, blending is a simple, efficient and scalable procedure for polymer synthesis, especially in tailoring the properties of synthetic polymers with more than two components. Moreover, the blend is energy-saving and labor-saving without commercial disadvantages of copolymer. Recently, many reports indicated the feasible applications of PLLA/PCL blends [5], [13], [17]–[22]. However, PLLA/PCL blend polymer has not been hitherto electrospun to NFS for a detailed research.

The human adipose-derived stem cells (*h*ASCs) are regarded as a forefront reservoir of seed-cell in tissue engineering [2], [23]. In comparison to other available sources (*eg.* embryonic stem cells and bone marrow-derived stem cells), *h*ASCs can be isolated from human subcutaneous adipose tissue discarded in liposuction lipoaspirate and surgeries which are performed everyday all around the world [24]. This merit avoids the ethical issue and highly invasive procedure. Because of simple harvesting procedure, abundant quantity, little immunity, low morbidity, and rapid expansion, *h*ASCs has paved an efficient path for tissue engineering. They are usually introduced to evaluate the biocompatibility of biomaterial by uniform seeding, continuous growth, and then differentiating into target cell lineage, which are all the basic principles for a desirable scaffold. There have been several reports on electrospun polymeric scaffolds fabricated with *h*ASCs [8], [25], [26]. Here, the *h*ASCs are appointed to investigate the biocompatibility of the PLLA and PCL blend fiber prepared by electrospinning method.

In this study, we manipulate electrospinning to prepare NFS of PLLA/PCL blend in different weight ratios (3/1, 2/1 and 1/1 of PLLA/PCL). The morphology, porosity, degradation, ATR-FTIR analysis, stress-stain assay and inflammatory reaction are investigated. To evaluate whether the NFS is biocompatible to substitute the natural ECM, *h*ASCs are seeded on NFS with various ratios for a comparative study on proliferation, attachment, viability, and differentiation.

## Materials and Methods

### Materials

Poly (L-lactide) (PLLA) with an average molecular weight of *M_w_*  = 100,000 was purchased from Shenzhen Guanghua Co. Ltd., China. Poly (ε-caprolactone) (PCL) with an average molecular weight of *Mw*  = 1,000 was purchased from Slovey Co., Korea. The two solvents of chloroform and methanol were analytical grade and used to dissolve PLLA and PCL without further purification. All cell culture medium and reagents were purchased from Goodtime Bio-Technology Co. Ltd., China.

### Preparation of NFS by Electrospinning

The PLLA and PCL blend solutions in three weight ratios (3/1, 2/1, 1/1) were submitted to electrospinning. Our previous study indicated that PLLA/PCL blends with 1/1 or more PLLA had acceptable electrospinability and the obtained fibers were uniform [27]. Briefly, two polymers were dissolved in chloroform and methanol (∼3/1 volume ratio) to the concentration of 10 wt%, followed by magnetic stirring for 4 h, and added into a 2 ml syringe with the mass flow rate of 1.0 ml/h. The electrospinning was run in an electrical field of 20 kV with the positive electrode inserting into the solution and negative electrode connecting with a grounded aluminum foil as collector which was positioned opposite with a perpendicular distance of 15 cm to the needle tip of syringe. All processing steps were performed at room temperature. The obtained material films were dried in vacuum and stored in desiccators for the following uses.

### Characterization of NFS

#### Morphology

The morphology of the electrospun NFS was observed by scanning electron microscopy (SEM, Phillips, Quanta 400, Netherland). The specimen was coated with a thin layer of gold before SEM observation. The fiber average diameter (AD) was statistically calculated by analyzing 20 fibers at random.

#### Porosity

The porosity (ε) is measured by density bottle and calculated according to the following equation: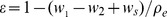 [28]. Briefly, the density bottle was filled with ethanol (density *ρ_e_*) and weighed (*w_1_*). Then a specimen of 3×3 mm^2^ (weight *w_s_*) was immersed into the bottle. The density bottle was filled with ethanol again and weighed (*w_2_*).

#### Degradation

The degradation *in vitro* of NFS was tested using a modified weight method according to the previous report [29]. Briefly, the NFS film was resized to 50×50 mm^2^ and weighed, and then immersed into a closed bottle containing phosphate-buffered saline (PBS, 0.01 mol/l, pH 7.4) at 37°C. After incubation at 37°C for indicated time intervals, the specimen was taken out, washed with distilled water, dried, and weighed again. The degree of degradation was evaluated by the percentages of dried weights after incubation dividing by primary weight.

#### ATR-FTIR analysis

An attenuated total reﬂection Fourier transform infrared (ATR-FTIR) spectrometer (Bruker, Equinox 55, Germany) was used to obtain the spectra of the NFS.

#### Stress-stain assay

Mechanical property of the electrospun NFS was tested by a universal testing machine (SUNS, CMT-4104, China) equipped with a 10 N load cell. The specimen was cut to rectangular shape of 80×20 mm^2^ and the tensile test was operated under a crosshead speed of 10 mm/min at ambient conditions.

#### Histological biocompatibility

The NFS specimen was implanted into subcutaneous pockets of Sprague-Dawley (SD) rats to investigate the histological response as a graft. The experimental protocol of animal was approved by the Ethics Committee of Tongji medical college according to national guideline for laboratory animals. Nine male adult SD rats (weight range 146g∼174g) were used with 3 rats for each kind of NFS. Under inhalation anesthesia and asepsis, each rat was subcutaneously implanted with NFS film (20×20 mm^2^) in dorsal pocket. At the time intervals of 1, 2, 4 weeks after implantation, the NFS specimen with connective tissues around was resected and treated by hematoxylin and eosin (H.E.) staining to detect the expression of CD68, the representative factor for macrophagocyte-mediated inflammation, using rabbit polyclonal antibodies against rat and goat anti-rabbit IgG/biotin (Bioss Biotech, China).

### Isolation and Culture of *h*ASCs

*h*ASCs were isolated from human subcutaneous adipose tissue discarded from 5 outpatients treated by plastic liposuction operation (females, mean age 34.3 year, range 24∼36 year) using the method reported by Zuk *et al* [24]. The Medical Ethics Committee of Tongji Medical College of Huazhong University of Science and Technology approved the protocol in this study and written informed consents were obtained from all patients. Briefly, adipose tissues were minced extensively, washed twice, and digested with 0.075% type I collagenase. The *h*ASCs were isolated by subsequent twice centrifugation and resuspension, and then plated in a 25-cm^2^ culture flask (Corning, USA) with culture medium (CM) consisted of Dulbecco’s modified Eagle’s medium (DMEM) (Gibco, USA) supplemented with 10% fetal bovine serum (FBS) (Hyclone, USA), 100 unit/ml of penicillin (Sigma-Aldrich, USA) and 100 µg/ml of streptomycin (Sigma-Aldrich, USA). The medium was refreshed every 3 days. The *h*ASCs were passaged by 0.25% trypsin/0.05% EDTA (Sigma-Aldrich, USA) and their 4th to 6th passages were used in the following study.

### Fabrication of *h*ASCs and NFS

The NFS specimens were resized into disks of 5 mm in diameter, sterilized in 75% alcohol for 2 h, washed in PBS for 1 min, and placed at the bottoms of wells in 96-well tissue culture plates (Costar, USA) with 5 repeated wells for each kind of NFS in a plate line. The *h*ASCs were released by trypsin/EDTA, regulated to a density of 5×10^5^ cells/ml in CM, and then pelleted into the wells with NFS at 200 µl per well to fabricate *h*ASCs-NFS constructs. The constructs were maintained at 5% CO_2_, 37°C and medium was replaced every 3 days. The *h*ASCs in conventional culture were treated identically in the rest wells of the plate without NFS as control.

### Cellular Responses to NFS

#### Cell morphology

The morphology of *h*ASCs-NFS constructs were photographed by a CK40 inverted phase contrast microscope (Olympus, Japan) at random location at 1, 3 and 7 days after fabrication. The light microscopy-observed specimens were further observed by SEM. Before SEM, the constructs were washed with PBS, fixed in 2.5% glutaraldehyde for 20 min, dehydrated in a series of graded concentrations of ethanol, dried in vacuum and gold-sputtered.

#### Cell proliferation

The proliferation curves were generated by MTT assay (Beyotime Biotech, China). From 1∼7 days after *h*ASCs-NFS fabrication, the average numbers from 5 repeated wells of *h*ASCs, with or without NFS, were reported as absorbance values at 490 nm.

#### Cell attachment

The percentage of attached *h*ASCs on NFS was tested with the aid of MTT assay. After fabrication, the *h*ASCs-NFS constructs were incubated for 6 h or 24 h, washed twice with PBS for 30 s, and transferred to new wells. The percentage was calculated using the absorbance value of construct specimen divided by that of control *h*ASCs without NFS.

#### Cell viability

The LIVE/DEAD Viability/Cytotoxicity Assay Kit (Molecular Probes, USA) was applied to distinguish viable cells from dead cells by a two-color (green and red) fluorescence label. At day 7, the construct specimens were transferred to a new 96-well cell culture plate, processed according to the manufacture’s instruction, and observed under a confocal laser scanning microscope (Olympus, FV500, Japan). The percentage of living cells, which was obtained from 3 randomly-chosen views, was designated to represent cell viability.

#### Multilineage differentiation

The multipotential including osteogenesis, chondrogenesis and adipogenesis (see protocols in Table 1) was investigated by histochemistry staining, quantitative real time polymerase chain reaction (Real-time PCR), and western blotting (WB).

**Table 1:** The protocol of multilineage differentiation.

Lineage	Medium Components	Induction Period	Medium RefreshFrequency
Osteogenesis	culture medium supplemented with 0.01 mM 1,25-dihydroxyvitamin D3, 50 mM ascorbate-2- phosphate, 10 mM b-glycerophosphate, 1% penicillin/streptomycin	2 weeks	twice a week
Chondrogenesis	DMEM supplemented with 1% FBS, 37.5 µg/ml ascorbate-2-phosphate, 1% penicillin/streptomycin, ITS premix, 10 ng/ml TGF-β1	2 weeks	twice a week
Adipogenesis	culture medium supplemented with 0.5mM 3-isobutyl-1-methylxanthine (IBMX), 0.2mM indomethacin 0.1 μM dexamethasone, 10 μM insulin, 1% penicillin/streptomycin	2 weeks	twice a week

For histochemistry staining, Von Kossa (Polysciences, Germany), Collagen II (Sigma-Aldrich, USA), and Oil red “O” (Sigma-Aldrich, USA) staining were assigned respectively to evaluate *h*ASCs differentiation to osteogenic, chondrogenic and adipogenic lineage on NFS. All steps were in accordance with the instructions of reagent manufactures.

For Real-time PCR and WB, the experimental procedures and reagents were identical with our previous study [30]. Briefly, the complementary DNA was reversely transcribed from the total RNA extracted by the TRIzol reagent (Invitrogen, USA) from the differentiated *h*ASCs in NFS using a PrimeScript® RT Reagent Kit (Takara, Japan). The reaction was manipulated according to the SYBR® Premix Ex TaqTM (Takara, Japan) instruction. Differently, the primers (Sangon, China) used in this study of osteocalcin (bone formation marker), aggrecan (chondrogenesis marker), peroxisome proliferator-activated receptor-γ (PPARγ, expressed mainly in adipose tissue), and GAPDH (a housekeeping gene) as internal control were changed to target human specificity (see primer sequences in Table 2). Moreover, the proteins were extracted from *h*ASCs-NFS constructs by a cocktail kit containing 100 mg/L phenylmethyl- sulfonyl fluoride (Goodtime, China) and were employed in equal amount determined with a BCA assay (Invitrogen, USA). After electrophoretical separation and transfer steps, the proteins were blocked with 5% fat-free milk and then incubated with primary antibodies (Boster, China) respectively targeting to osteocalcin, aggrecan, PPARγ, and GAPDH. The bands were exposed in a darkroom in the presence of HRP-conjugated IgG as a secondary antibody (Boster, China).

**Table 2:** The sequence of primers for Real time-PCR.

mRNA	Sequence	Product size (bp)
osteocalcin	forward: gcagcgaggtagtgaagagac	**241**
	reverse: aaagaagggtgcctggagag	
aggrecan	forward: ctctgggttttcgtgactctg	**246**
	reverse: cagcactacctccttctccttg	
PPARγ	forward: gccttttggtgactttatgga	**175**
	reverse: ggcttgtagcaggttgtcttg	
GAPDH	forward: agaaggctggggctcatttg	**258**
	reverse: aggggccatccacagtcttc	

### Statistical Analysis

All experiments were run in triplicate, if not stated specially, for the NFS of each weight ratio and control group and all data were expressed as mean ± standard deviation. The results were statistically analyzed by SPSS 13.0 software using ANOVA and student *t* test. Differences were accepted as statistical significance at a level of p<0.05.

## Results and Discussion

The electrospinning process is a promising way for providing non-woven, nanometer-scale fibrous structure [8], [9]. Early efforts of electrospinning PLLA and PCL synthesis as tissue- engineered biomaterials focused largely on their copolymers [3], [14]–[16]. Compared to copolymer, a significant advantage of blending polymer is the ability to modify the property as required with simple procedure and accurately-controlled manipulation [13], [21]. Our previous work once verified the electrospinability of PLLA/PCL blend and practical parameters in our electrospinning system. Only one fiber specimen prepared by the confirmed parameters was submitted to biocompatibility investigations with rabbit ASCs [27]. In this study, the PLLA/PCL blends prepared by electrospinning in different weigh ratios were introduced to human ASCs to explore the ratio-biocompatibility effect for humanizing potential in tissue engineering.

### Effects of Blending Weight Ratio on NFS Properties

#### Morphology

**Figure 1:**
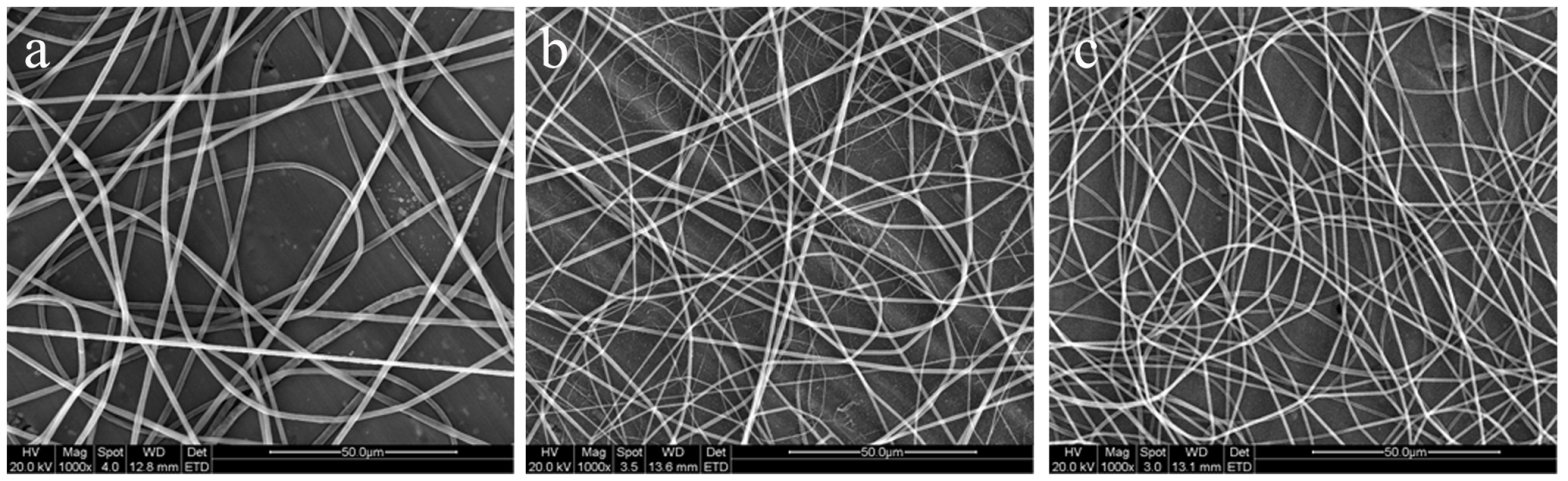
SEM micrographs of electrospun NFS in different weight ratios: (a) 3/1 PLLA/PCL, (b) 2/1 PLLA/PCL, and (c) 1/1 PLLA/PCL. Scale bar  = 50 μm. At the condition of mass flow rate of 1.0 ml/h, electrical field of 20 kV and syringe needle tip-to-collector distance of 15 cm, the PLLA/PCL blends of varied blending weight ratios were electrospun into fibers. The AD of fibers decreased with the increasing PCL content in the blends.

The micrographs of NFS of 3/1, 2/1, and 1/1 blending weight ratio were shown by SEM in Figure 1. The AD of fibers varied from 1.25±0.65 μm (Figure 1a), to 968±57 nm (Figure 1b), to 682±24 nm (Figure 1c) with the increased PCL content in the blends. Morphologically, with PCL component increasing in the blend, the fiber diameter decreased, the shape turned uniform, the porosity increased, the three-dimensional space augmented, and hydrolytic weight reduced. These mainly caused by the low viscosity of PCL. The more PCL content leaded to the lower solution viscosity and larger fibrous cross-linked range.

#### Porosity

The porosity measured by density bottle revealed about 78±7%, 79±8%, and 82±6% space in 3 types of NFS, respectively. As the PCL content increased, the porosity gradually enlarged. However, there was no significant difference in the porosity data among 3 NFS groups (p>0.05).

#### Degradation

**Figure 2:**
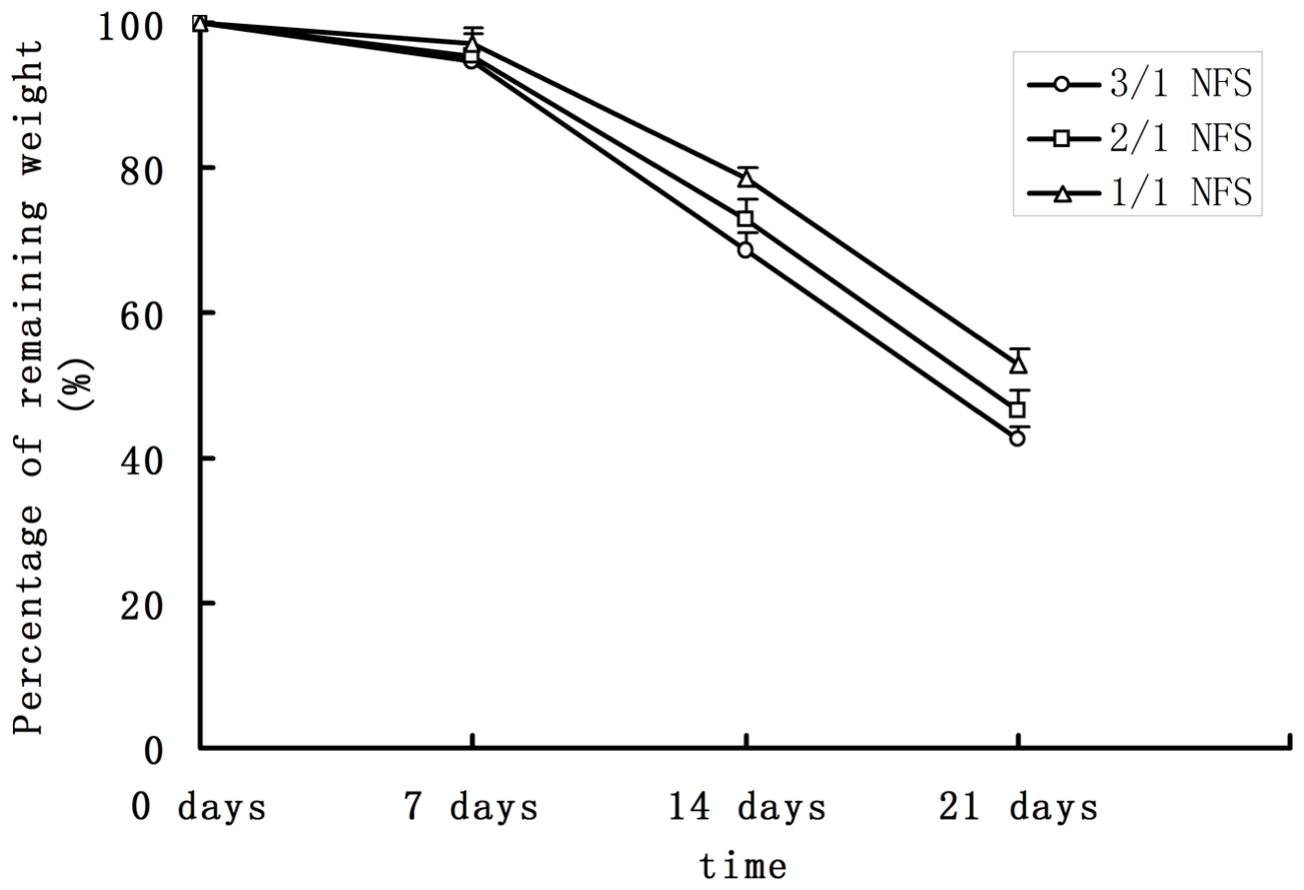
Degradation curve of different NFS *in vitro*. As mentioned in the introduction paragraph, the PLLA degrades faster than PCL. Thus, the degradation curve of 3/1 NFS was below the curves of 2/1 and 1/1 NFS.

The percentages of remaining weight after degrading *in vitro* were shown in Figure 2. After 7 d, specimens degraded in a slow rate. The remaining weights were 94.7±2.0% (3/1 NFS), 95.4±3.3% (2/1 NFS), and 97.3±3.9% (1/1 NFS), respectively. One week later, the remaining weights decreased dramatically to 68.7±1.4% (3/1 NFS), 72.9±2.7% (2/1 NFS) and 78.7±2.5% (1/1 NFS), with the statistically significant difference (p<0.05). Here, more remaining weight percentage meant less degradation rate. Our data indicated that the NFS of less PLLA had slower degradation rate. This was consistent with our knowledge that PLLA was highly-degraded as previously introduced.

#### ATR-FTIR

**Figure 3:**
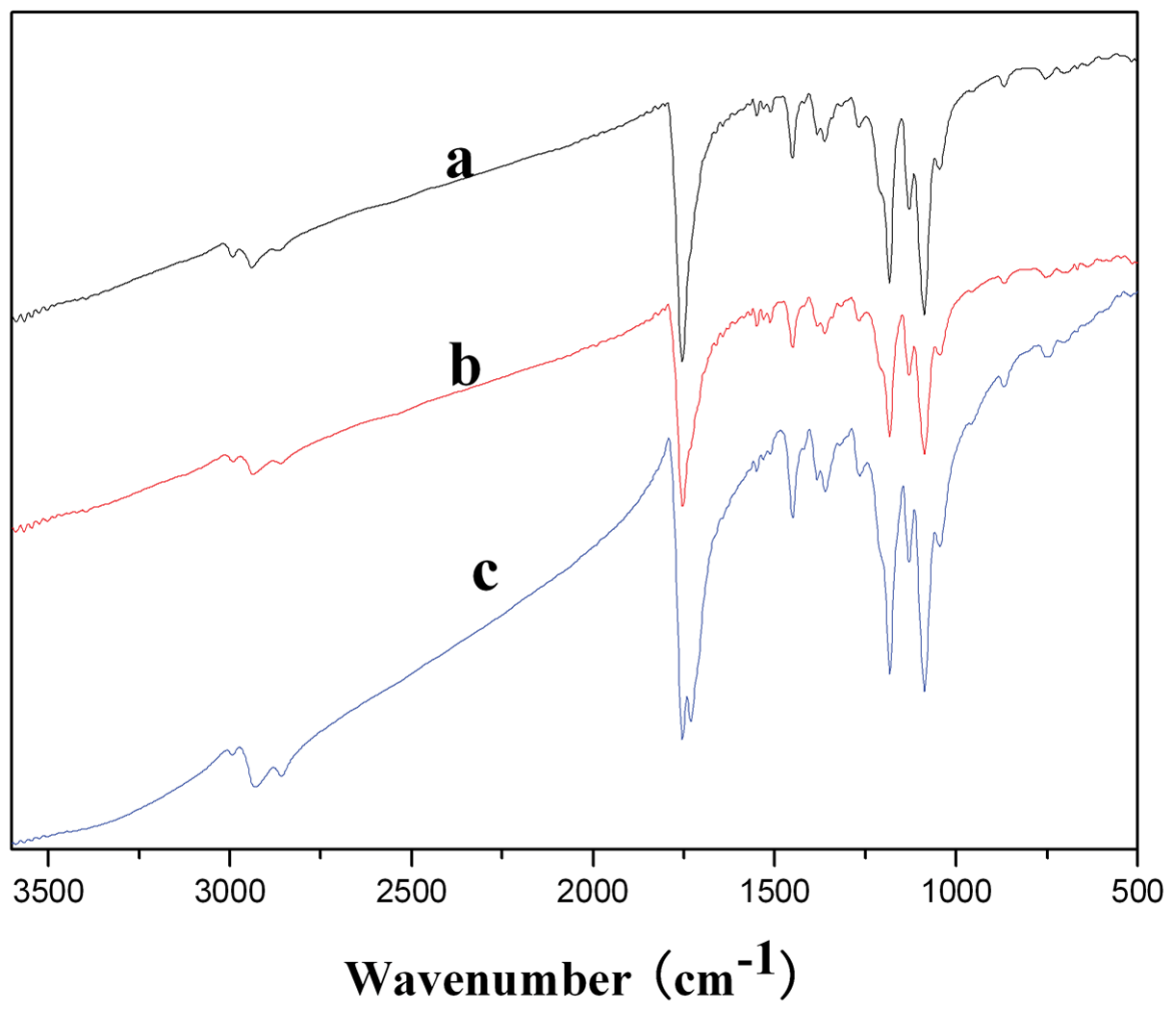
FTIR spectra of different NFS: (a) 3/1 NFS, (b) 2/1 NFS, and (c) 1/1 NFS. The 1/1 ratio NFS group exhibited carbonyl stretching (C = O) at 1754 cm^−1^ (due to PLLA) and 1731 cm^−1^ (due to PCL), while other NFS group gave a single peak at 1754 cm^−1^.

A set of representative FTIR spectra of NFS was shown in Figure 3. FTIR spectra of the 1/1 ratio NFS exhibited carbonyl stretching (C = O) at 1754 cm^−1^ (due to PLLA), 1731 cm^−1^ (due to PCL) while NFS of other ratio gave a single peak at 1754 cm^−1^. The carbonyl stretch region was wider with more PCL component. This may be due to merging of two peaks into one as a result of blending of two polymers.

#### Stress-stain curve

**Figure 4:**
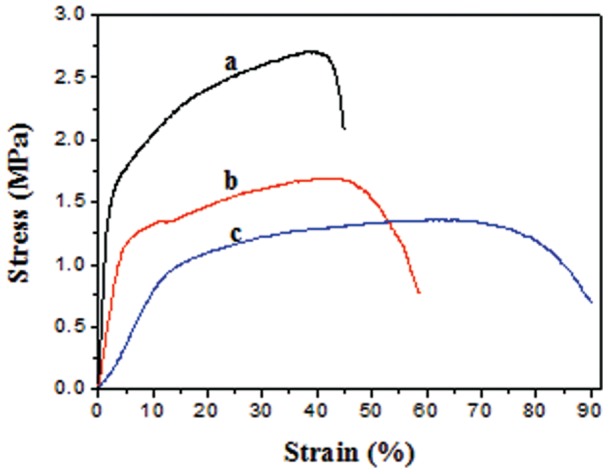
Tensile stress–strain curves of the electrospun NFS: (a) 3/1 NFS, (b) 2/1 NFS, and (c) 1/1 NFS. As the PCL content increased, Young’s modulus and tensile strength decrease. However, its elongation at break increases.

The stress–strain curves were shown in Figure 4. Corresponding tensile strength, Young’s modulus, and elongation-at-break were listed in Table 3. As the PCL content increased, Young’s modulus and tensile strength decreased to 6.5±0.7 MPa and 1.2±0.1 MPa from 40.1±0.8 MPa and 2.7±0.1 MPa. However, its elongation at break increased by 113%, i.e., its elongation at break increased from 40.5±8.6% (3/1 NFS) to 85.6±7.2% (1/1 NFS). The results suggested that the rubbery PCL performed as a molecular coil, while the brittle PLLA as strong domains. This further confirmed the advantages of flexibility and deformability in blending PLLA and PCL process.

**Table 3:** Mechanical properties of the electrospun PLLA/PCL NFS.

Mechanical Properties	3/1 NFS	2/1 NFS	1/1 NFS
Young’s modulus (MPa)	40.1±0.8	25.3±0.4	6.5±0.7
Tensile strength (MPa)	2.7±0.1	1.7±0.1	1.2±0.1
Elongation at break (%)	40.5±8.6	54.5±6.4	85.6±7.2

#### Histological biocompatibility

**Figure 5:**
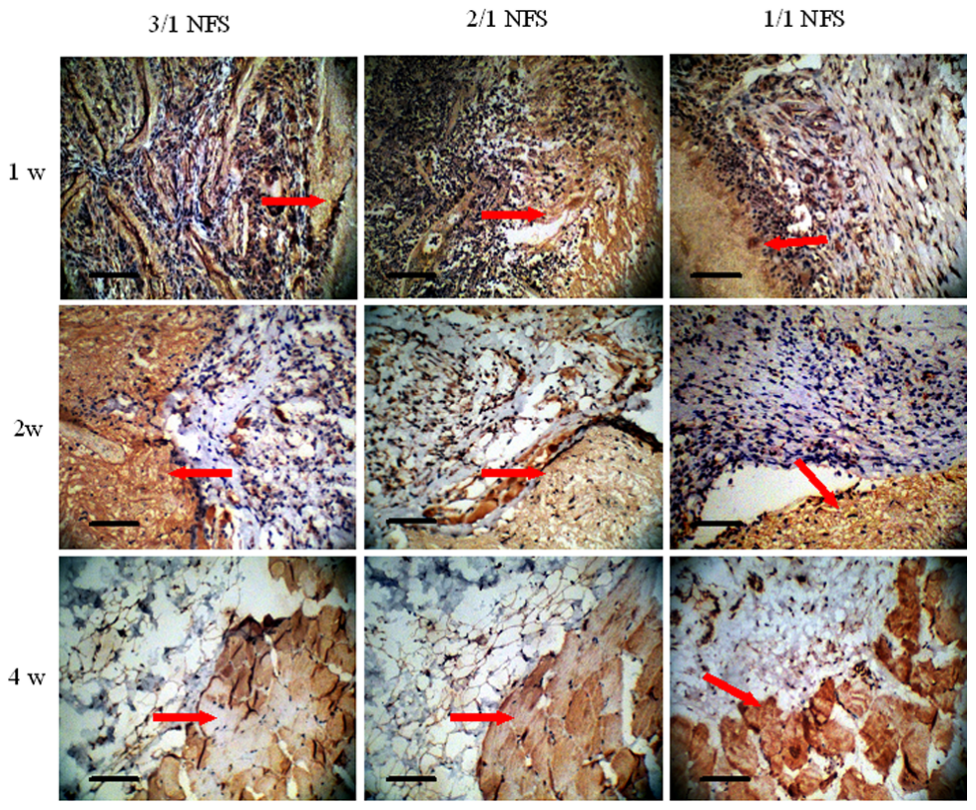
The expression of CD68 by immunohistochemistry in inflammatory reaction caused by NFS. Scale bar  = 100 μm. CD68 is a representative factor which expresses positively in non-specific inflammation mediated by macrophagocyte. The red arrows indicate the NFS debris embedded in paraffin sections which stained to be light brown. At 1w after NFS implanting into subcutaneous pockets of SD rats, the all three type NFS were surrounded by a large number of CD68^+^ macrophagocytes showed purple. As the time passed by, the purple macrophagocytes decreased, and then vanished, which meant the inflammation reaction ended.

Figure 5 showed the expression of CD68 was at a high level at 1st week and decreased gradually later. At the 4th week, the macrophagocyte almost vanished. No obvious difference was seen among the 3 groups of NFS. The period of inflammatory reaction triggered by NFS as foreign body was littler longer (4 weeks) than normal (near 1 week) in body. Whatever, 4-week imflammatory procedure was acceptable as foreign body transplantation.

### Effects of Blending Weight Ratio on *h*ASCs’ Response to NFS

#### Morphology

**Figure 6:**
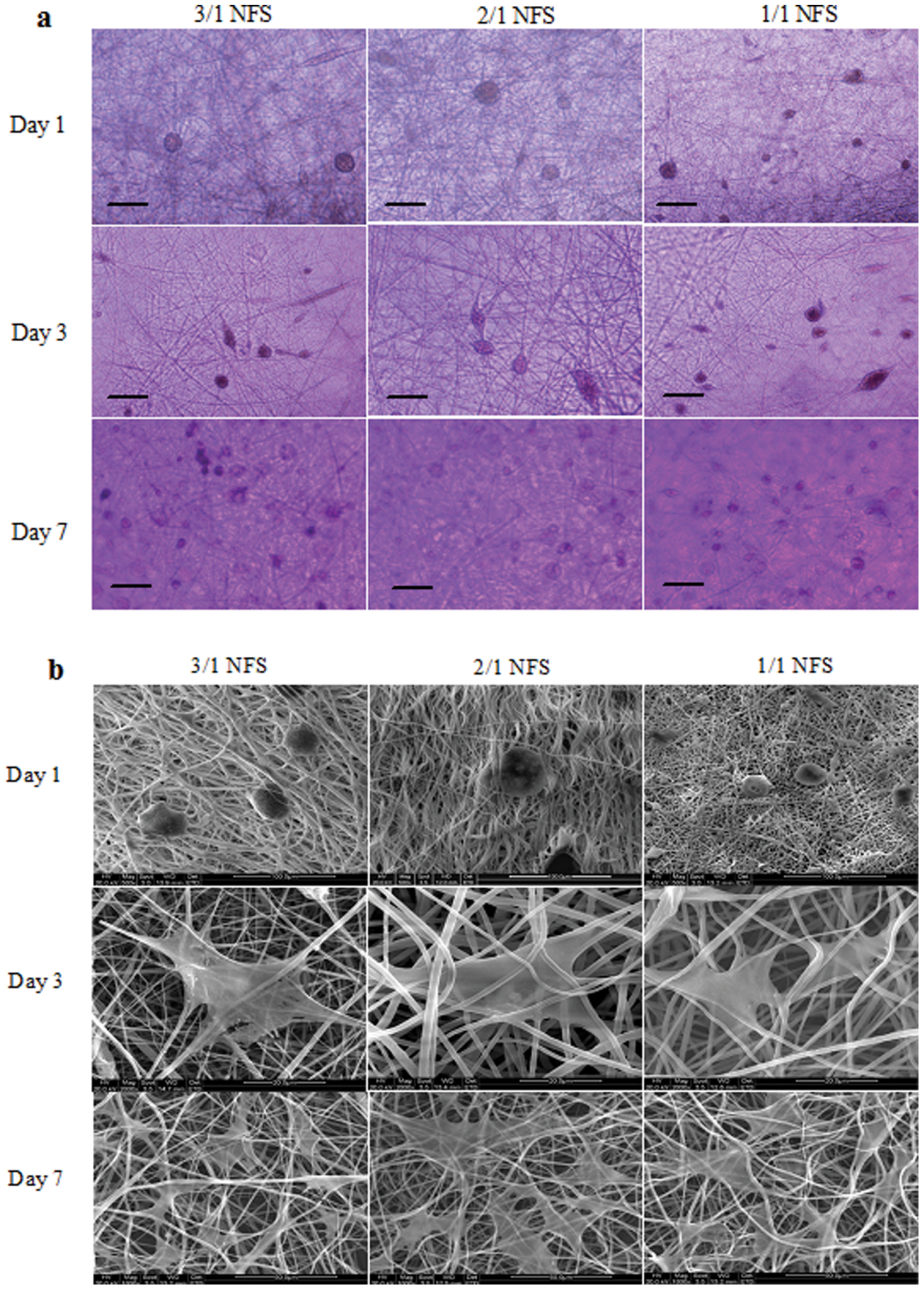
The morphology of *h*ASCs on different NFS: (a) under light microscopy. Scale bar  = 100 μm; (b) under SEM. Scale bar  = 50 μm. Both in (a) and (b), the *h*ASCs showed round spots because they released from culture flasks by trypsin on day 1 after seeding in NFS. On day 3, the *h*ASCs extend their bodies attaching on the NFS fibers and proliferated in the nanofibrous space. Seen in (a), on day 7, the number of *h*ASCs increased in all 3 NFS. In (b), the *h*ASCs showed like white film in irregular shape attaching on the fibers around and grew in number from day 3 to 7.

Both of light microscopy (Figure 6A) and SEM images (Figure 6B) of *h*ASCs-NFS constructs were shown in Figure 6. Obviously, 1/1 NFS mimicked ECM better than the other two groups due to a higher cell density on it observed by both two methods.

#### Proliferation

**Figure 7:**
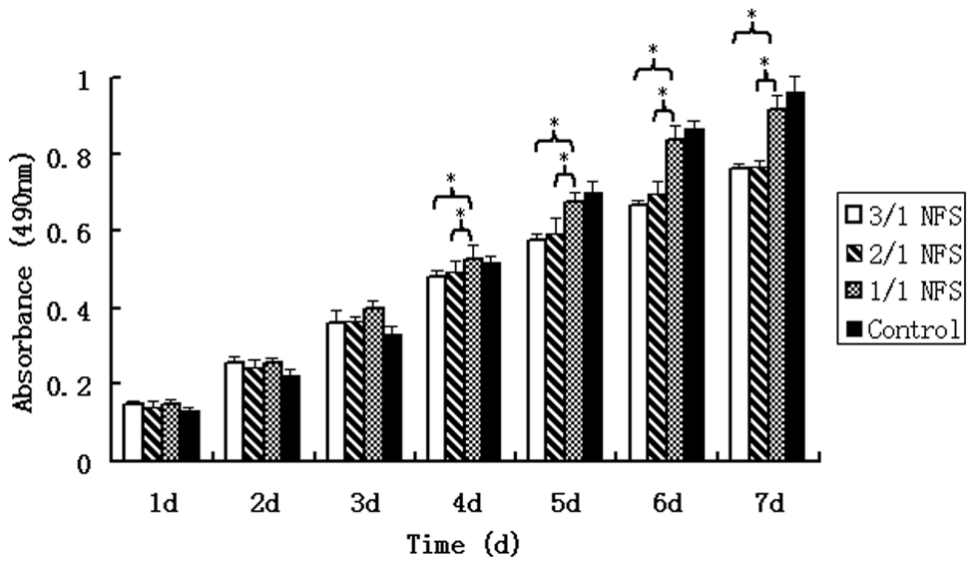
MTT assay of *h*ASCs on different NFS. From day 1 to 7 after fabrication of *h*ASCs and NFS, the cell numbers expressed as absorbance of MTT assay increased continuously. From day 4, the *h*ASCs number in 1/1 NFS was significantly higher than 3/1 and 2/1 NFS (both p<0.05). But the difference between 1/1 NFS group and control group was not analyzed to be a statistical significance (p>0.05). * indicated there was a statistically significant difference between the two groups connected by a by a bracket.

In Figure 7, the absorbance value reflected indirectly the corresponding cell number. Interestingly, the *h*ASCs’ numbers from 3 NFS groups all exceeded that of control group in the first 3 days. The reason was suggested that the three-dimensional NFS provided more surface area for *h*ASCs in the early stage. During the synthetic the accumulated carbon dioxide increased the acid degree of the culture medium, which broke the balance of micro-environment during cell growth. The 1/1 NFS degraded slowest, so it underwent the least toxic substance. From the day 4, the *h*ASCs in 1/1 NFS group grew faster than 3/1 and 2/1 NFS group (both p<0.05 compared by 1/1 NFS *vs.* 3/1 and 1/1 NFS *vs.* 2/1 NFS). However, the difference between 1/1 NFS group and control group was analyzed to be no statistical significance (p<0.05) though mostly the controlled cell numbers were more than 1/1 NFS group. The result implied that the 1/1 NFS was biocompatible enough to replace the natural ECM.

#### Attachment

**Figure 8:**
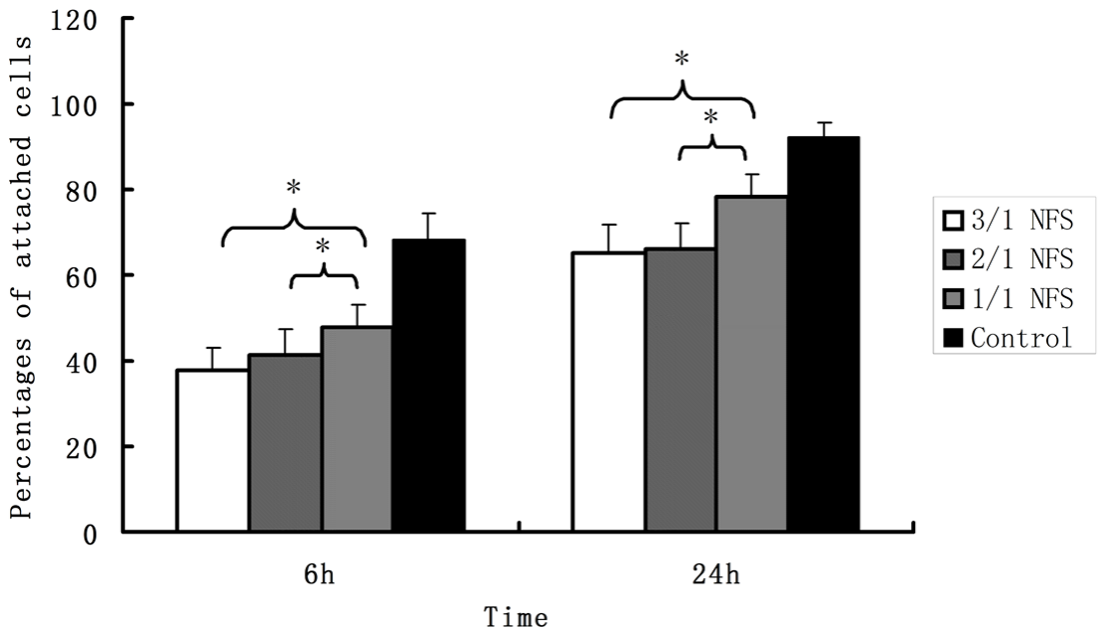
The percentages of attached *h*ASCs on different NFS. The attachment percentage of 1/1 NFS group was obviously more than 3/1 NFS and 2/1 NFS whether at 6 h or 24 h. (all p<0.05 when pairwise compared). The control *h*ASCs, having the highest attachment percentage without doubt, was not necessary to be included in the statistical analysis already. * indicated there was a statistically significant difference between the two groups connected by a bracket.

The percentages of attached *h*ASCs calculated by the recorded absorbance values of MTT assay were shown in Figure 8. In 1/1 NFS group, there were 47.8±5.1% and 78.4±4.9% cells remained respectively at two time points, obviously more than 3/1 NFS (37.8±5.2% and 65.3±6.5%) (p<0.05), and 2/1 NFS (41.1±6.3% and 66.2±6.1%) (p<0.05). The control *h*ASCs, without doubt, had the highest attachment percentage.

#### Viability

**Figure 9:**
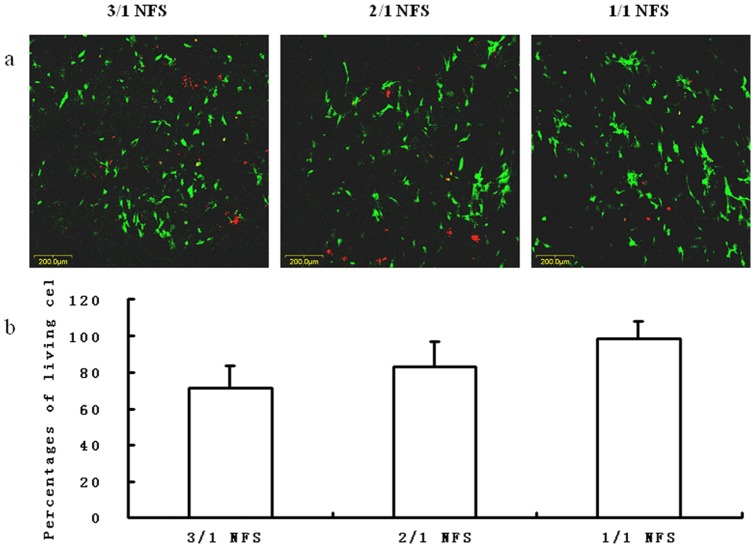
The LIVE/DEAD staining of *h*ASCs on different NFS: (a) images under confocal laser scanning microscopy at day 7. The green points represented living cells and the red points were dead cells. Scale bar  = 200 μm. (b) percentages of living cells at day 7. The percentage was calculated by the counting number obtained from 3 randomly-chosen views. No significant difference was detected among the 3 NFS groups (p>0.05).

In Figure 9, living and dead *h*ASCs were probed by green and red fluorescence (Figure 9a). At day 7 after fabrication, the percentages of living cells in random views were calculated to form a histogram (Figure 9b). Although there was a larger number of living *h*ASCs on 1/1 NFS than either of 3/1 and 2/1 NFS, no significant difference was detected among 3 NFS groups (p>0.05).

#### Multilineage differentiation

**Figure 10:**
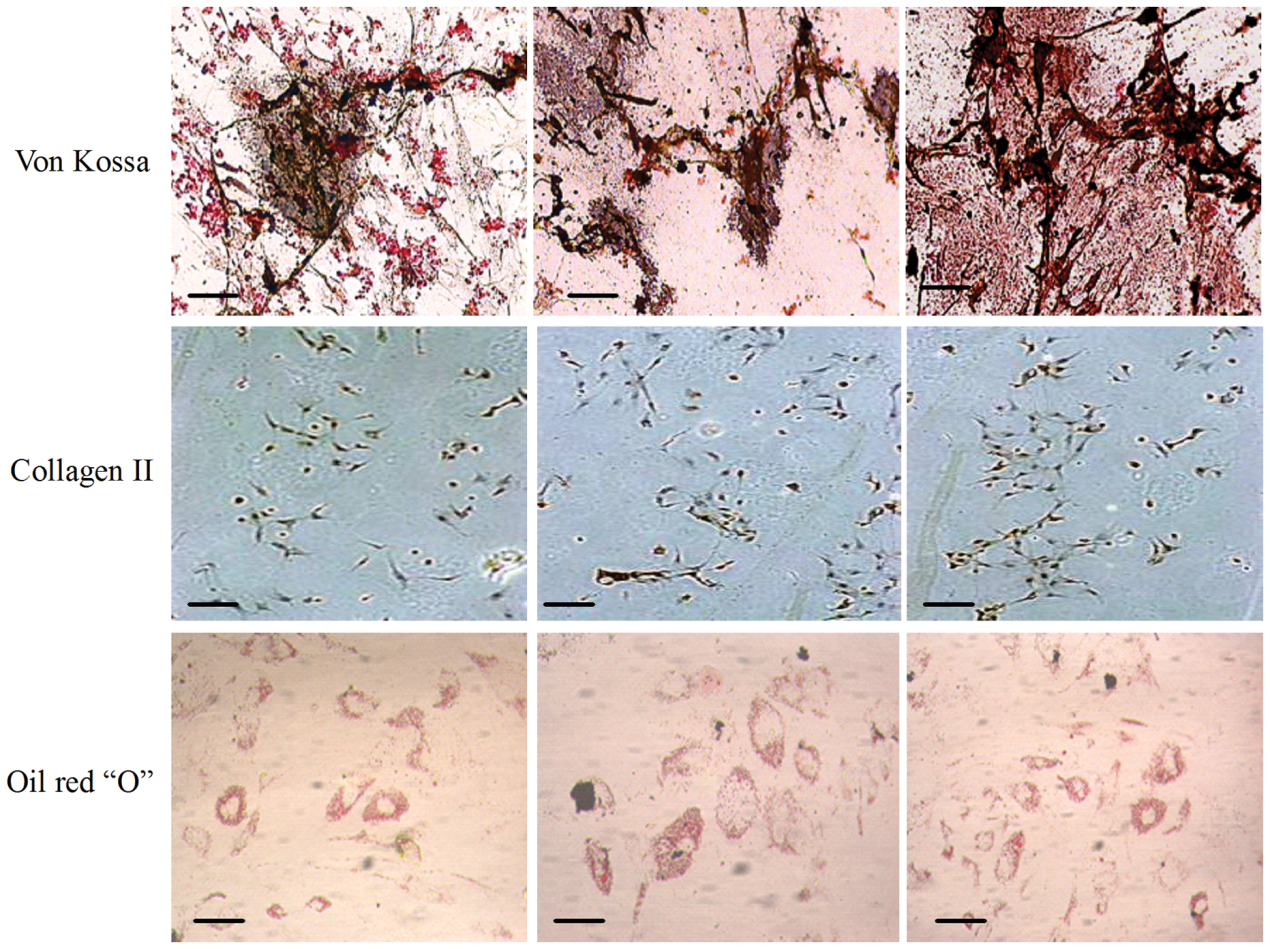
The histochemical staining of multilineage differentiated *h*ASCs on different NFS. Scale bar  = 100 μm. In Von Kassa staining, the accumulated calcium released from the osteogenetic *h*ASCs was stained brown. In Collagen II staining, the chondrogenetic *h*ASCs were stained brown, too. In Oil red “O” staining, the oil drops kept in the adipogenic *h*ASCs were stained red.

**Figure 11:**
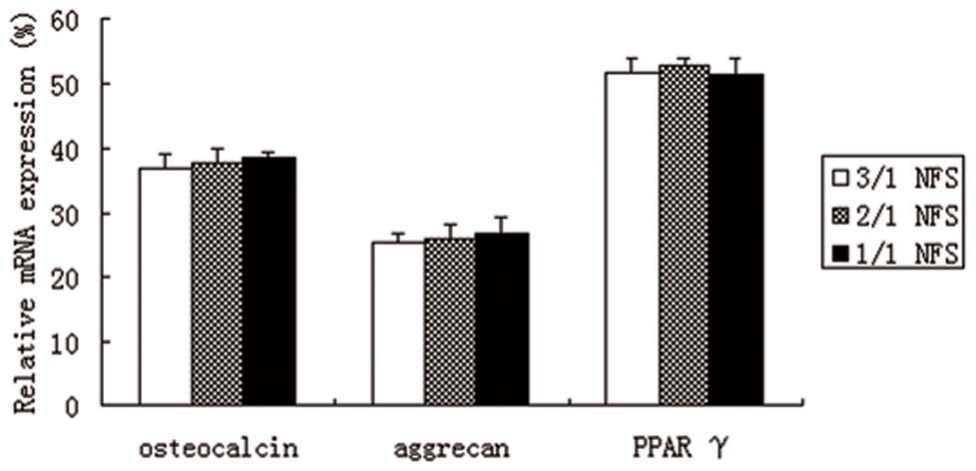
The relative expression of target genes of multilineage differentiation of *h*ASCs on different NFS. No difference was statistically analyzed to be significant (all p>0.05).

**Figure 12:**
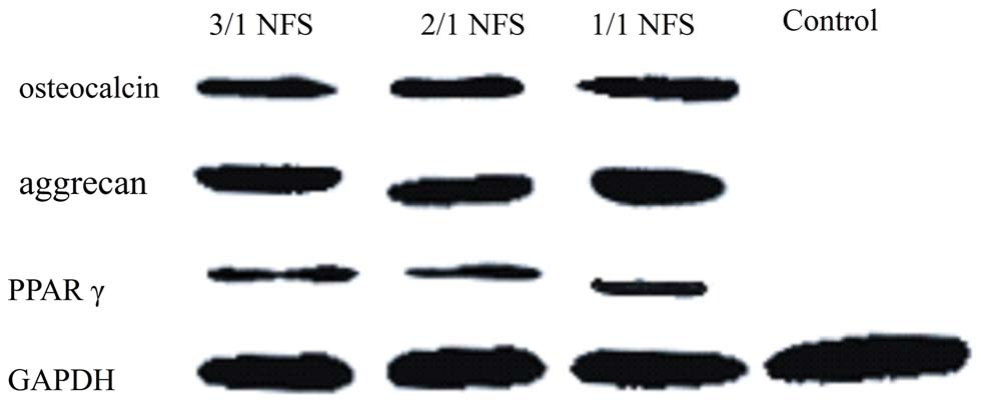
The western blotting bands of target proteins from differentiated *h*ASCs on different NFS.

The *h*ASCs-NFS constructs were induced to osteogenesis, chondrogenesis, and adipogenesis *in vitro.* As shown in Figure 10, Von Kossa, Collagen II, and Oil red “O” staining characterized respectively the calcium accumulation, collagen production, and oil depot. After the Real-time PCR results in Figure 11 and WB bands in Figure 12 verified further for the expression of specific genes and proteins. No significant difference was found among constructs of 3 NFS groups (all p>0.05). In the differentiation investigation committed to three lineages, histochemistry staining, Real time-PCR, and Western Blot were comprehensively analyzed to draw a conclusion that different blending weight ratios of such NFS had little effect on the *h*ASCs’ multipotential. Thus, the polymeric structure and degradation rate directly affected cellular proliferation, attachment, and viability, indirectly exerting influence on differentiation ability.

## Conclusions

In the research of biomaterial characterization, as expected, the synthesized NFS showed integrated properties between pure PLLA and pure PCL, similarly with their copolymer [3], [14], [16]. When seeded by *h*ASCs, it was obvious that the uniform structure and thin fiber of 1/1 NFS offered the more appropriate shelter for *h*ASCs to replace ECM.

To sum up, the PLLA and PCL, as the mostly used biodegradable polymers in tissue engineering, could be blended and electrospun into nanofibrous scaffold, and showed commendable properties. The trend was confirmed that more PCL component in blends brought better biocompatibility of NFS. However, as reported before [27], the fact was also witnessed that only blends with more PLLA could be electrospun into ideal fibers. Consequently, the weight ratio of 1/1 was the balance point between the electrospinability and biocompatibility. This newly-synthesized blend polymer will draw remarkable attention for its desirable characterization and convenient procedure.
